# Miltefosine-Lopinavir Combination Therapy Against *Leishmania infantum* Infection: *In vitro* and *in vivo* Approaches

**DOI:** 10.3389/fcimb.2019.00229

**Published:** 2019-06-28

**Authors:** Karina M. Rebello, Valter V. Andrade-Neto, Claudia Regina B. Gomes, Marcos Vinícius N. de Souza, Marta H. Branquinha, André L. S. Santos, Eduardo Caio Torres-Santos, Claudia M. d'Avila-Levy

**Affiliations:** ^1^Laboratório de Estudos Integrados em Protozoologia, Instituto Oswaldo Cruz, Fundação Oswaldo Cruz (FIOCRUZ), Rio de Janeiro, Brazil; ^2^Laboratório de Bioquímica de Tripanosomatídeos, Instituto Oswaldo Cruz, FIOCRUZ, Rio de Janeiro, Brazil; ^3^Laboratório de Síntese de Substâncias no Combate a Doenças Tropicais, Farmanguinhos, FIOCRUZ, Rio de Janeiro, Brazil; ^4^Laboratório de Estudos Avançados de Microrganismos Emergentes e Resistentes, Instituto de Microbiologia Paulo de Góes, UFRJ, Rio de Janeiro, Brazil

**Keywords:** chemotherapy, co-infection, HIV, HIV-PI, leishmaniasis, treatment

## Abstract

Concurrently, leishmaniasis and AIDS are global public health issues and the overlap between these diseases adds additional treats to the management of co-infected patients. Lopinavir (LPV) has a well characterized anti-HIV and leishmanicidal action, and to analyze its combined action with miltefosine (MFS) could help to envisage strategies to the management of co-infected patients. Here, we evaluate the interaction between LPV and MFS against *Leishmania infantum* infection by *in vitro* and *in vivo* approaches. The effect of the compounds alone or in association was assessed for 72 h in mouse peritoneal macrophages infected with *L. infantum* by the determination of the IC_50_s and FICIs. Subsequently, mice were orally treated twice daily during 5 days with the compounds alone or in association and evaluated after 30 days. The *in vitro* assays revealed an IC_50_ of 0.24 μM and 9.89 μM of MFS and LPV, respectively, and an additive effect of the compounds (FICI 1.28). The *in vivo* assays revealed that LPV alone reduced the parasite load in the spleen and liver by 52 and 40%, respectively. The combined treatment of infected BALB/c mice revealed that the compounds alone required at least two times higher doses than when administered in association to virtually eliminate the parasite. Mice plasma biochemical parameters assessed revealed that the combined therapy did not present any relevant hepatotoxicity. In conclusion, the association of MFS with LPV allowed a reduction in each compound concentration to achieve the same outcome in the treatment of visceral leishmaniasis. Although a pronounced synergistic effect was not evidenced, it does not discard that such combination could be useful in humans co-infected with HIV and *Leishmania* parasites.

## Introduction

Visceral leishmaniasis (VL), also known as kala-azar, is a vector-borne disseminated protozoan infection caused by species of the *Leishmania donovani* complex (Lukes et al., 2007; Burza et al., 2018). It is a an important but neglected tropical disease that occurs worldwide (Ready, 2014). In 2015, more than 90% of VL occurred in only seven countries: Brazil, Ethiopia, India, Kenya, Somalia, South Sudan, and Sudan (Burza et al., 2018). Notwithstanding, VL remains prevalent in more than 60 countries worldwide (Burza et al., 2018).

VL is an opportunist disease in human immunodeficiency virus (HIV) infected patients and this co-infection is one of the major challenges for VL control (Alvar et al., 2008). The re-emergence of VL in Europe in the 1990's was caused by immigration and HIV infection worsened the scenario (Agostoni et al., 1998). Since then, co-infection cases have been reported in 35 countries worldwide (Lindoso et al., 2016), being more prevalent in the East Africa region, especially Ethiopia (Van Griensven et al., 2014b; Yimer et al., 2014), in Brazil (Nascimento et al., 2011; Lima et al., 2018), and in India (Burza et al., 2014; Singh, 2014). VL promotes an increase in viral load and accelerates the clinical progression of acquired immunodeficiency syndrome (AIDS), thereby reducing the life quality and expectancy of these patients. On the other hand, HIV co-infection significantly increases the risk of progression to VL disease in asymptomatic or subclinical individuals (Alvar et al., 2008; Ezra et al., 2010; Adriaensen et al., 2017). Indeed, it has been shown that the immunological status of HIV patients is favorable for the multiplication of *Leishmania* parasites (Adriaensen et al., 2017). Thus, both pathogens exert synergistic detrimental effect on the immune response of co-infected patients (Ezra et al., 2010).

Despite VL/HIV co-infection representing a significant public health burden, the current therapies are inefficient, and an effective treatment is remaining a challenge (Ritmeijer et al., 2011; Sinha et al., 2011; Van Griensven et al., 2014a). VL/HIV co-infection cases have higher rates of treatment failure, greater susceptibility to drug toxicity and higher lethality and relapse than in VL infected patients without HIV infection (Monge-Maillo et al., 2014; Van Griensven, 2014; Van Griensven et al., 2014c). The advent of the highly active antiretroviral therapy (HAART) improved the life quality, increased the life expectancy of HIV patients, as well as promoted a substantial reduction on the incidence of opportunistic infections (Crabtree-Ramírez et al., 2016; Lindoso et al., 2016). Particularly, HIV-aspartyl peptidase inhibitors (HIV-PIs) have been described as a powerful *in vitro* antiproliferative agents against several opportunistic pathogens (Pozio and Morales, 2005; Trudel et al., 2008; Santos et al., 2009; Santos, 2010; Lindoso et al., 2016). Previous data from our research group demonstrated that nelfinavir is an effective antileishmanial agent against promastigotes of several *Leishmania* species (Santos et al., 2013), as well as that lopinavir (LPV) affects *Leishmania*-macrophage interaction (Santos et al., 2009).

The combination therapy may be an interesting strategy to deal with the co-infection. Previous studies have shown that drug association can be very effective, reducing side effects, decreasing the induction of resistance, and allowing the prescription of lower doses to achieve the same outcome (Perron et al., 2012; Stone et al., 2014; Trinconi et al., 2014; Sun et al., 2016). Driven by the necessity of finding alternative therapeutic strategies for VL/HIV co-infection, we evaluated the combination treatment with LPV, an HIV-PI, and miltefosine (MFS) in *L. infantum* infection. Our results suggest that LPV- MFS combination therapy can be effective in the treatment of VL/HIV co-infected patients and provides data that can help to guide a possible therapeutic strategy in VL/HIV co-infection.

## Materials and Methods

### Drugs and Chemicals

LPV was synthesized in the Laboratory of Chemical Synthesis, Farmanguinhos, FIOCRUZ. MFS, heat inactivated fetal bovine serum (FBS), RPMI-1640 medium, streptomycin, penicillin, hemin, D-biotin, adenine, folic acid, AlamarBlue®, and dimethylsulfoxide (DMSO) were purchased from Sigma Aldrich Chemical (St. Louis, MO, USA). Drugs were prepared in DMSO, aliquoted, and kept at −20°C until use. All other reagents were analytical grade or superior.

### Parasites

*Leishmania infantum* (strain MHOM/MA/67/ITMAP-263) was cultivated at 26°C in RPMI medium supplemented with 10% FBS, streptomycin (100 μg/mL), penicillin (100 U/mL), hemin (5 mg/mL), D-biotin (0.2 mg/mL), adenine (4 mg/mL), and folic acid (0.5 mg/mL).

### Experimental Animals and Ethics Statement

BALB/c mice (female, 6–8 weeks old) were obtained from the Institute of Science and Technology in Biomodels (ICTB-FIOCRUZ). Mice were housed five per cage and maintained in standard environmental conditions (12:12 h light:dark cycle at 22 ± 2°C) with access to food and water *ad libitum*.

### Cytotoxicity Assay

The AlamarBlue® assay was used to determine the cytotoxicity of LPV and MFS in uninfected mouse macrophages. Resident peritoneal macrophages from BALB/c mice were seeded at 1 × 10^6^ cells/mL in 200 μL supplemented RPMI into 96 well-plates at 37°C in 5% CO_2_ for 4 h for adherence. Then, the plates were gently washed two times with PBS (phosphate buffered saline, 150 mM NaCl, 20 mM phosphate buffer, pH 7.2) to remove non-adherent cells, and treated with 2-fold serial dilutions of LPV and MFS concentration ranging from 400 to 3.125 μM and 40 to 0.3125 μM, respectively. After 72 h, AlamarBlue® was added to the macrophage cultures to a final concentration of 10% v/v, and the plates were then incubated at 37°C for additional 4 h. The absorbance was measured at excitation/emission of 560/590 nm (Kulshrestha et al., 2013; Cunha-Júnior et al., 2017). The results were expressed as the percentage of viable cells compared to the control cells treated with the highest DMSO dose used to dissolve the compounds.

### Evaluation of *in vitro* Antileishmanial Activity

Resident peritoneal macrophages from BALB/c mice were resuspended in supplemented RPMI medium. 8 × 10^5^ cells/well were plated in eight chamber Lab-Tek chambers (Nunc, Roskilde, Denmark). *L. infantum* promastigotes collected at the stationary phase were washed three times in PBS (3,000 × g for 10 min) and added to adherent cells at a parasite/macrophage ratio of 5:1 and incubated for 4 h at 37°C in 5% CO_2_. Next, free promastigotes were removed by washing with RPMI medium and the macrophages were incubated with LPV alone or in combination with MFS at 37°C for 72 h. The solutions were prepared in proportions of 5:0, 4:1, 3:2, 2:3, 1:4, and 0:5 of LPV and MFS drugs, respectively, which were serially diluted (base 2) six times. The initial drug concentrations were 25 and 2 μM of LPV and MFS, respectively. LPV initial concentration was the highest non cytotoxic to macrophages, while for MFS, we chose the most potent (non-cytotoxic) concentration that did not completely eliminate parasites from macrophages in the single compound assays. Three independent experiments, in triplicate, were performed for each drug combination and susceptibility assay.

Finally, the slides were fixed, stained with Panoptic and the amastigotes were counted using light microscopy. The infection rate was calculated using the formula: (% of infected macrophages × average number of amastigotes per macrophage). Control experiments were performed with infected macrophages incubated with DMSO at the highest dose used to dissolve the compounds. The 50% inhibitory concentration (IC_50_), i.e., the minimum drug concentration that caused a 50% reduction in infection rate in comparison with that in control infection without the compound, was obtained by non-linear regression using GraphPad Prism software. Each point was tested in duplicate with three biological replicates.

### Fractional Inhibitory Concentration Determination and Isobologram Construction

The four fractional inhibitory concentration indexes (FICIs) of LPV, derived from association curves, were calculated using the following equation: concentration of LPV in each association curve (4:1, 3:2, 2:3, 1:4) able to inhibit 50% of the parasite growth/ IC_50_ of LPV alone. The same formula was applied to MFS. The sum FICIs (ΣFICIs) were calculated as FICI of LPV plus FICI of MFS and the arithmetic mean of the FICIs obtained was compared to the reference values and reported as synergism (FICI ≤ 0.5), antagonism (FICI ≥ 4.0) and additive effect of the compounds (0.5 < FICI < 4.0) (Odds, 2003). The interaction between drugs was expressed graphically as an isobologram.

### Mice Infection and Treatment

BALB/c mice were infected intraperitoneally with 1.0 × 10^8^ stationary-phase *L*. *infantum* promastigotes. After 7 days, animals were treated by oral gavage twice daily for 5 days with a 12 h interval between doses following the dosages described below (Katsuno et al., 2015; Cunha-Júnior et al., 2017). Thirty days post infection, the animals were euthanized, and the spleen and liver were aseptically removed, weighed, and homogenized in supplemented RPMI medium. The parasite load was estimated by limiting dilution assay (LDA) (Buffet et al., 1995). Plasma biochemical parameters investigated were aspartate aminotransferase (AST) and alanine aminotransferase (ALT), creatinine (CREA), urea, total bilirubin and cholesterol, which were measured by the Program of Technological Development in Tools for Heath PDTIS-Fiocruz.

### Therapeutic Scheme

The animals were treated with either MFS, LPV or the combination of both drugs by the oral route twice daily (at 12 h intervals) for 5 days (Katsuno et al., 2015) at day seven post-infection (Cunha-Júnior et al., 2016). Animals were divided into 13 groups, as follows: (0) Control, non-infected and non-treated (CNI); (1) Control, PBS with 1% DMSO, infected and non-treated (CI); subsequently, all groups correspond to infected and treated mice, as follows: MFS at 15.4 mg/kg (2), 7.7 mg/kg (3), 3.85 mg/kg (4); LPV at 493.2 mg/kg (5), 246.6 mg/kg (6); MFS + LPV, respectively, at 7.7 mg/kg + 493.2 mg/kg (7), 7.7 mg/kg + 246.6 mg/kg (8), 3.85 mg/kg + 493.2 (9), 3.85 mg/kg + 246.6 mg/kg (10), 1.92 mg/kg + 493.2 mg/kg (11), 1.92 mg/kg + 246.6 mg/kg (12). Each group was composed of at least five mice, and the experiment was repeated three times, independently.

### Statistical Analyses

The results are presented as means ± standard deviation (SD) or standard error of the mean (SEM) of replicates samples from at least two independent assays. Paired comparisons between groups were carried out by Student's *t*-test or analysis. *P*-values equal or >0.05 were considered statistically significant.

## Results

### Evaluation of the *in vitro* Efficacy of LPV-MFS Combination

**Figure 1:**
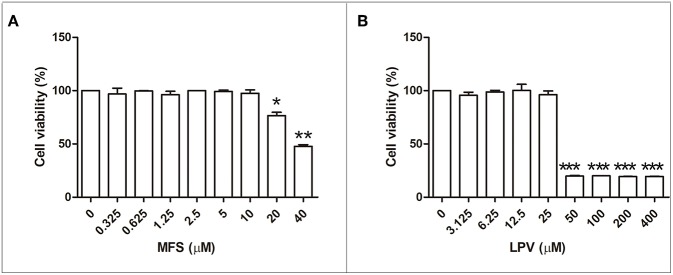
Citotoxicity of MFS and LPV to peritoneal macrophages. Cells (1 × 10^6^ cells/mL) were incubated in 96 well plates for 72 h in the presence of MFS **(A)** and LPV **(B)** at different concentrations. The viability of macrophages was assessed by using the Alamar blue assay. Data represent the mean (±SD) of three independent experiments. *P < 0.05, **P < 0.01, and ^***^P < 0.001.

**Figure 2:**
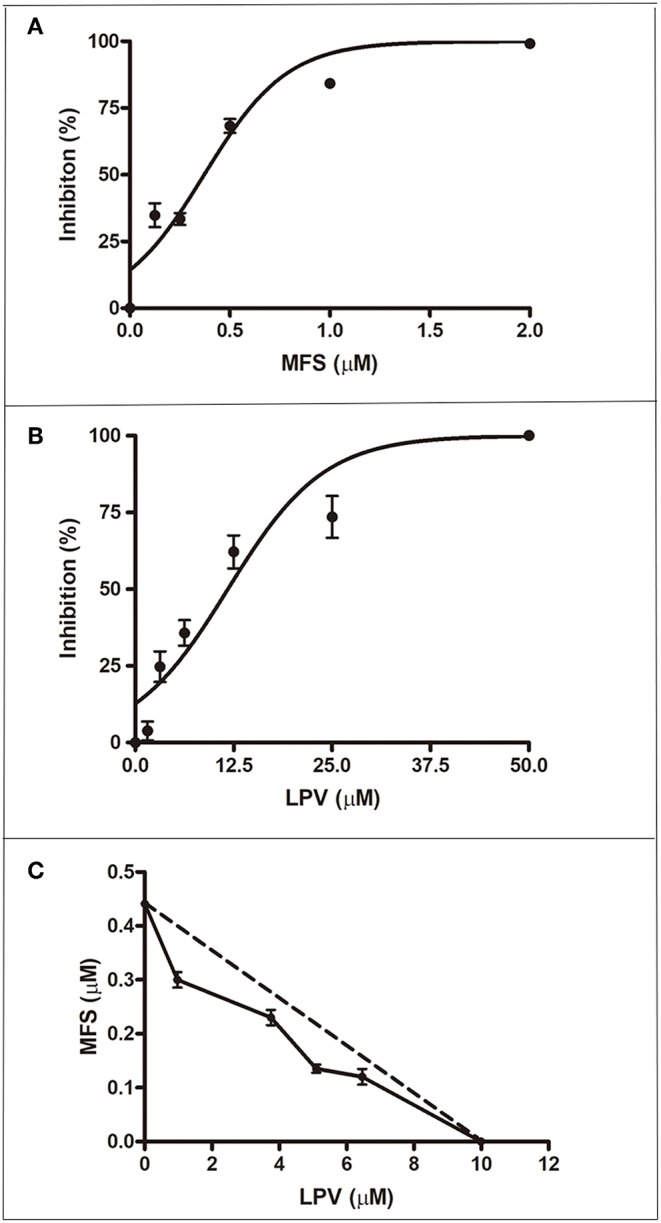
Antiamastigote activity of MFS, LPV, and their combination. Peritoneal macrophages infected with *L. infantum* were treated with MFS **(A)**, LPV **(B)**, or both drugs associated **(C)** for 72 h at 37°C. **(C)** Isobologram analysis of antiamastigote activity of drugs combined in several proportions. Each plotted point in the isobolograms is the IC_50_ of the drug alone or in combination. The straight dashed line represents the theoretical line of additivity for each combination. Data are representative of three independent experiments and values are expressed in mean ± SD in **(A,B)** and ± SEM in **(C)**.

First, we aimed to determine the highest drug concentration of each compound that was not cytotoxic to macrophages under the assayed conditions, which were 25 and 20 μM for LPV and MFS, respectively (Figure 1). Then, the antiamastigote activity was evaluated for the drugs alone or associated in several proportions, as described in the materials and methods section. The antileishmanial activity of LPV and MFS was confirmed against intracellular *L. infantum* amastigotes, with IC_50_ of 9.89 ± 0.2 and 0.44 ± 0.3 μM, respectively (Figures 2A,B). The resulting effect of the drugs association was evaluated graphically by plotting the IC_50_ of the compounds alone or in combination as an isobologram (Figure 2C). In addition, the FICI value for each drug combination was calculated. The ${\;}_{\bar{x}}$FICI was1.28 ± 0.24, indicating an additive interaction (Odds, 2003) between LPV and MFS (Table S1). Furthermore, none of the concentrations of the drugs tested in combination induced any significant toxicity as assessed by AlamarBlue assay (data not shown).

### *In vivo* Efficacy of Drugs in the Murine VL Model

**Figure 3:**
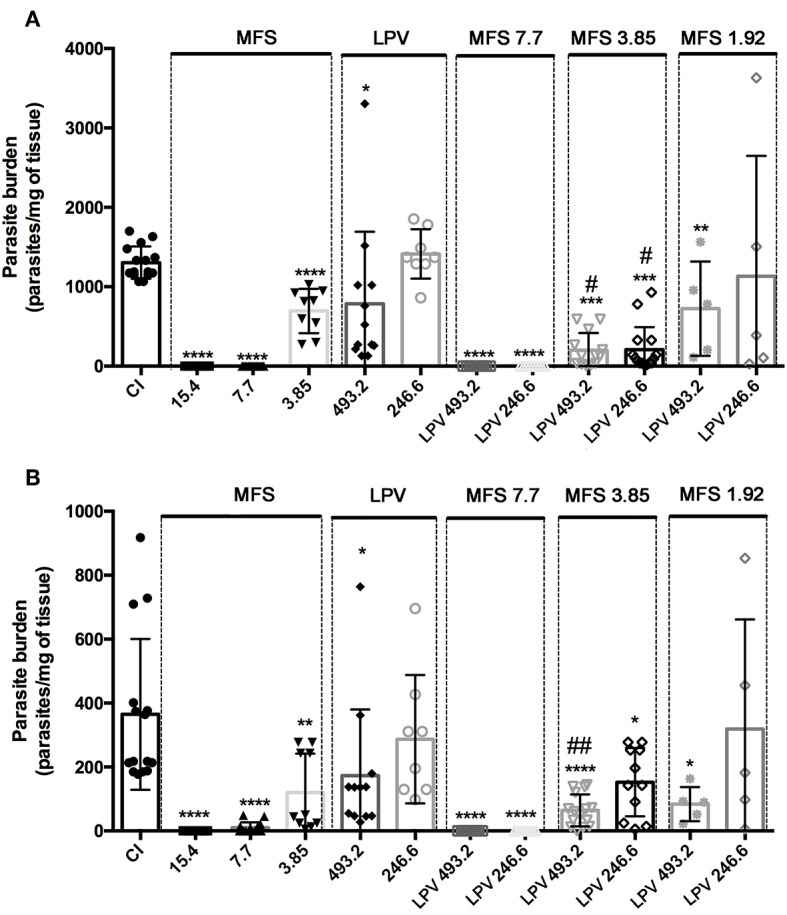
Efficacy of MFS alone or in combination with LPV in *L. infantum in vivo* infection. Evaluation of hepatic **(A)** and splenic parasite burden **(B)** 30 days post-infection. CI, infected control. Animals were treated in day 7 post-infection by oral gavage twice daily for 5 days with a 12 h interval between doses. Data are presented as the mean ± SD. ^*^P < 0.05; ^**^P < 0.01; ^****^P < 0.0001 versus CI. ^#^*P* < 0.05 vs. MFS 3.85; ^##^*P* < 0.01 vs. MFS 3.85.

For *in vivo* assays, *L. infantum* infected BALB/c mice were treated with MFS at 15.4, 7.7, 3.85, and 1.92 mg per kg of body weight alone or in combination with LPV at 493.2 and 246.6 mg per kg of body weight. In MFS-treated mice, the hepatic and splenic amastigote loads were completed suppressed by 7.7 and 15.4 drug doses, respectively (Figure 3). This compound at 3.85 mg/kg promoted a significant reduction in the mean of parasitic load in liver (46.7% ± 7.1) and spleen (67% ± 10.5) (Figure 3). In LPV-treated mice the hepatic and splenic amastigote loads were statistically significant reduced by the treatment with 493.2 mg/kg to 40% (±20) and 52% (±16.4), respectively (Figure 3). In conclusion, as expected, MFS alone was able to reduce parasite burden compared to untreated infected control. Conversely, LPV at the highest dose tested presented a reduction in the parasite load that is not negligible.

Concerning the drug association at first, we showed that LPV did not exert any deleterious effect on MFS action by adding it at 493.2 and 246.6 mg/kg to a MFS dose that virtually eliminated the parasite (7.7 mg/kg) (Figure 3). Then, we tested the effect of LPV at the same concentrations on a MFS lower dose (3.85 mg/kg), which alone presented only an intermediary effect. The hepatic parasitic load was reduced in 70.15% (±4.6) and 71% (±5.6) in relation to single MFS treatment with 3.85 mg/kg or control infected mice, respectively, while no dose-response was observed between the two concentrations of LPV. In spleen, only the highest dose of LPV promoted a significant reduction (52.56% ± 16.4) in the parasite load, in relation to MFS alone (3.85 mg/kg). Finally, we analyzed the combination of MFS at 1.92 mg/kg with LPV at 493.2 and 246.6 mg/kg, this MFS dose has as a marginal effect based on the results at 3.85 mg/kg. The highest dose of LPV showed a significant reduction in hepatic (44.48% ± 20) and splenic (77% ± 6.5) parasite load (Figure 3).

**Figure 4:**
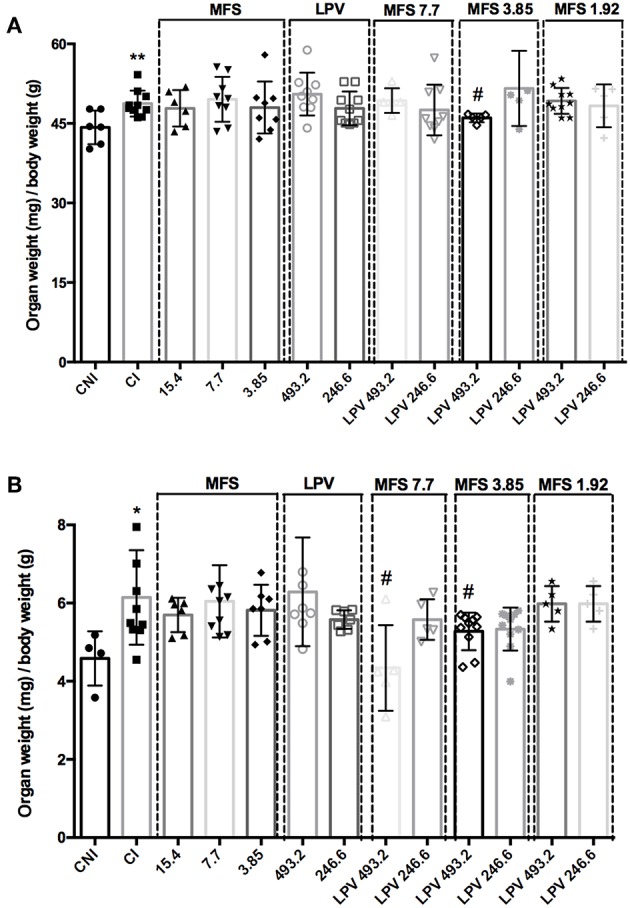
Relative organ weights of mice in different treatment with MFS alone or in combination with LPV. **(A)** Liver, **(B)** Spleen. Animals were treated in day 7 post-infection by oral gavage twice daily for 5 days with a 12 h interval between doses. The control group (CI) was treated with 1% DMSO in saline via the oral route. A non-infected and non-treated control group (CNI) was also evaluated. ^*^P < 0.05 vs. CNI; ^**^P < 0.01 vs. CNI; ^#^*P* < 0.05 vs. CI.

As expected, CI group presented a significant increase in spleen and liver relative weight when compared to CNI (Figure 4). A significant decrease in the liver weight in comparison to CI was only observed in the infected mice treated with 493.2/3.85 mg/kg of LPV/MFS (5.57% ± 0.82), while no statistical significance is observed when compared to CNI (Figure 4A), which indicates that a high dosage of LPV combined with MFS at 3.85 mg/kg reverted the liver weight to the levels of health individuals. The relative weight of spleen was significantly reduced in comparison to CI in mice treated with 493.2/7.7 and 493.2/3.85 mg/kg of LPV/MFS in 29.34% (SD 1.09) and 14.4% (SD 0.48), respectively (Figure 4B). Both treatments were able to revert the spleen weight to the levels of health individuals (Figure 4B). Finally, at the end of the treatment, the hepatic toxicity was evaluated by measuring the plasma levels of total bilirubin, ALT and AST (Table S2). No significant changes were observed in the bilirubin and ALT levels in comparison to CI. Increased circulation levels of AST were found in the serum of animals treated with MFS at 15.4 and 7.7 mg/kg, LPV at the highest dose (493.2 mg/kg) and in all combination doses. However, the AST values found for all doses tested are inside the normal range for mice (AST = 54-298 U/I) (Wege et al., 2012). The renal function was also evaluated and no significant changes for creatinine or urea levels in plasma of untreated and treated animals was observed (Table S2). Moreover, no differences were found in the serum cholesterol levels among all studied groups (Table S2). These data point out that the combined therapy did not present any relevant hepatotoxicity and impact on mice, under the assayed parameters.

## Discussion

The aim of this study was to evaluate the antileishmanial effect of LPV and MFS combination in an infection caused by *L. infantum* using *in vitro* and *in vivo* murine model of VL. The nature of interaction between the drugs was first determined as additive *in vitro*. This prompted us to assay the association *in vivo* in BALB/c mice infected with *L. infantum*, and the additive effect of both drugs observed *in vitro* was reproduced *in vivo*.

MFS interferes on *L. donovani* lipid metabolism, inducing an increase in sphingolipid and ergosterol content (Armitage et al., 2018). Sterol biosynthesis is a crucial pathway that leads to the production of ergosterol in *Leishmania* parasites, and therefore, it is an interesting chemotherapeutic target. Unlike mammalian cells, trypanosomatids synthesize C24-alkylated and ergostane-based sterols (Goad et al., 1984; McCall et al., 2015). Therefore, compounds that interfere with the sterol pathway are promising drugs in treating leishmaniasis. In addition, drugs that act synergistically on different points of the same pathway represent an attractive strategy for antimicrobial chemotherapy (Roberts et al., 2003; Andrade-Neto et al., 2016). In this sense, our group showed that LPV also alters the lipid composition on *L. amazonensis*, mainly interfering in sterol composition and causing a pronounced accumulation of cholesterol-ester in treated parasites (Rebello et al., 2018). Although the intracellular target or death mechanism of the HIV-PIs are not totally elucidated in trypanosomatids, it is also likely that they interact and inhibit trypanosomatids aspartyl peptidases (Santos et al., 2013; Castilho et al., 2018). Therefore, considering the multiple and diverse targets of each compound, the additive effect reported here was expected.

Recently, Valdivieso et al. reported the effects of the combined therapy with nelfinavir, another HIV-PI, and MFS in a murine infection by *L. infantum* (MCAN/ES/98/LLM-724) (Valdivieso et al., 2018). Mice experimentally infected were treated in day 15 by intraperitoneal injection of nelfinavir and MFS during 15 days, and then parasitemia was measured. This treatment is in high contrast to our scheme, which was oral gavage twice a day, for only 5 days in the seventh day post-infection. Mice were then sacrificed on day 30-post infection, therefore, before parasitemia was assessed, mice continued alive with no treatment during 18 days. Although Valdivieso et al. recently reported a more prominent combined effect, the treatment scheme reported here, strongly challenge the compounds efficacy, and the oral gavage, more closely resembles the administration route that is used for human patients, since MFS and HIV-PIs are oral drugs (Jha et al., 1999; Dorlo et al., 2012; Crabtree-Ramírez et al., 2016).

In the scenario of increasing cases of HIV/Leishmania co-infection, the data presented herein from oral-treated mice during only 5 days can help to guide the design of clinical trials for the specific management of co-infected individuals. The oral combined therapy of LPV-MFS was effective in reducing the parasite loads in animal models of visceral Leishmaniasis and boosted the effect of lower doses of MFS. We demonstrated the potential value of combining available oral and safer drugs as a promising strategy to treat VL/HIV co-infection patients, and envision the possibility of achieving the same treatment outcome with lower compounds dosages, which can prevent or delay drug resistance and reduce side effects in patients.

## Data Availability

All datasets generated for this study are included in the manuscript and/or the Supplementary Files.

## Ethics Statement

This study was carried out in accordance with the protocols approved by the Ethics Committee for Animal Use of the Instituto Oswaldo Cruz (CEUA-FIOCRUZ, license number: L-026/2015).

## Author Contributions

KR and VA-N performed experiments and data analysis. CG and MS provided reagents. MB and AS gave some advices for the work and provided valuable support with the writing. ET-S and Cd'A-L directed and coordinated the study. KR wrote the manuscript and all authors participated in editing it.

### Conflict of Interest Statement

The authors declare that the research was conducted in the absence of any commercial or financial relationships that could be construed as a potential conflict of interest.

## Supplementary Material

Table S1 IC_50_, FICI_50_, and ΣFICI_50_ of LPV-MFS combination against *L. infantum* intracellular amastigotes.

Table S2 Comparison of biochemical parameters between untreated (CI, control infected) and treated parasites with MFS or LPV administered alone or in combination. Levels of creatinine, urea, total bilirubin, cholesterol, AST (Alanine amino transferase), and ALT (Aspartate amino transferase) are shown. The data are mean ± SD of three independent experiments. ^*^*P* < 0.05, ^**^*P* < 0.01.
