# Supplementary material for: Miltefosine-Lopinavir Combination Therapy Against Leishmania infantum Infection: In vitro and in vivo Approaches
Source: Front Cell Infect Microbiol. 2019 Jun 28;9:229. doi: 10.3389/fcimb.2019.00229 (PMC6611157; doi:10.3389/fcimb.2019.00229)
Supplement: Table S1 — IC50, FICI50, and ΣFICI50 of LPV-MFS combination against L. infantum intracellular amastigotes. [file Table_1.DOCX]

|  | **Combination Rate**  (highest concentration) | | **Combinated drugs** | | | | | |
| --- | --- | --- | --- | --- | --- | --- | --- | --- |
|  |  |  | **IC_50_** (µM) | | **FICI_50_** | | **ΣFICI_50_** | **$\bar{x}$ΣFICI_50_** |
|  | **Lopinavir** | **Miltefosine** | **Lopinavir** | **Miltefosine** | **Lopinavir** | **Miltefosine** |  |  |
| **Proportion** | 0 | 5 (2µM) | --- | 0.44 ± 0.09 | --- | --- | --- | 1.28 ± 0.31 |
|  | 1 (5 µM) | 4 (1.6µM) | 0.96 ± 0.01 | 0.31 ± 0.07 | 0.10 ± 0.07 | 0.54 ± 0.04 | 0.64 ± 0.21 |  |
|  | 2 (10 µM) | 3 (1.2 µM) | 3.72 ± 0.03 | 0.24 ± 0.01 | 0.38 ± 0.07 | 1.83 ± 0.78 | 2.21 ± 0.99 |  |
|  | 3 (15 µM) | 2 (0.8 µM) | 5.07 ± 0.04 | 0.14 ± 0.08 | 0.51 ± 0.09 | 0.58 ± 0.11 | 1.09 ± 0.33 |  |
|  | 4 (20 µM) | 1 (0.4 µM) | 6.25 ± 0.30 | 0.13 ± 0.08 | 0.63 ± 0.12 | 0.54 ± 0.14 | 1.17 ± 0.27 |  |
|  | 5 (25µM) | 0 | 9.89 ± 0.15 | --- | --- | --- | --- |  |
| Data expressed as mean ± SEM. | | | | | | | | |
